# Supplementary material for: Genomics of Dwarfism in Italian Local Chicken Breeds
Source: Genes (Basel). 2023 Mar 3;14(3):633. doi: 10.3390/genes14030633 (PMC10047989; doi:10.3390/genes14030633)
Supplement: Supplementary file 1 [file genes-14-00633-s001.zip › Supplementary Material Table S2.pdf]

Table S2. Haplotypes derived from Haploview analysis and related to Figure S4. For each breed blocks and their frequencies in population are reported.

MUGELLESE (FIGURE S4a)	
**BLOCK 1**	**Allele frequency**
ATGACAGGTGATAAGGACCTCAAGGGTTGG	(0.437)
AGGACAGATCGGTAGGATTCCTCCAGTTGG	(0.271)
GGAGTGAAGGATAGAAGTTTTAAGAACCCA	(0.187)
AGAACAGATCGGTAGGATTCCTCCAGTTGG	(0.062)
AGGATGGAGGATAGGGGCCTCAAGGGTTGG	(0.021)
ATGACAGAGGAGTAGAGTTTTAAGAACCCA	(0.021)
**MERICANEL DELLA BRIANZA (FIGURE S4b)**	
**BLOCK 1**	**Allele frequency**
GGC	(0.542)
AAT	(0.312)
AGC	(0.125)
AGT	(0.021)
**BLOCK 2**	
AG	(0.604)
GT	(0.396)
**BLOCK 3**	
ACATGA	(0.521)
GTGGCT	(0.375)
GCAGGA	(0.083)
ACAGGA	(0.021)
**BLOCK 4**	
TCAACAAGCCACCG	(0.521)
CGGGTGCATGGATA	(0.375)
TGAACGCACCAACG	(0.062)
CCAGCACGCGAATG	(0.021)
CCAGTGCATGGATA	(0.021)
