# Supplementary figures and images for: Genomics of Dwarfism in Italian Local Chicken Breeds
Source: Genes (Basel). 2023 Mar 3;14(3):633. doi: 10.3390/genes14030633 (PMC10047989; doi:10.3390/genes14030633)

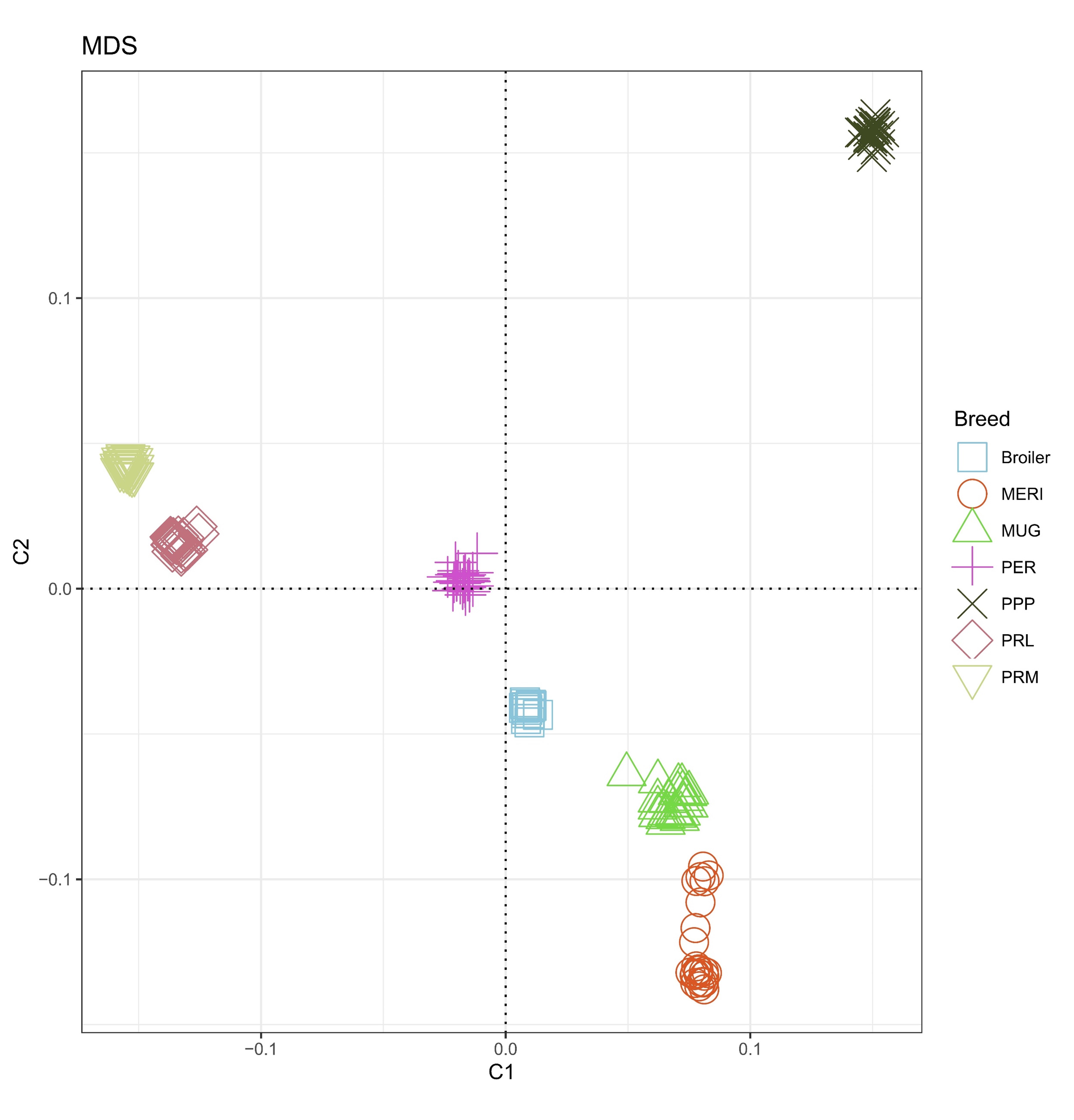

Supplement: Supplementary file 1 [file genes-14-00633-s001.zip › Supplementary Material Figure S1.jpg]

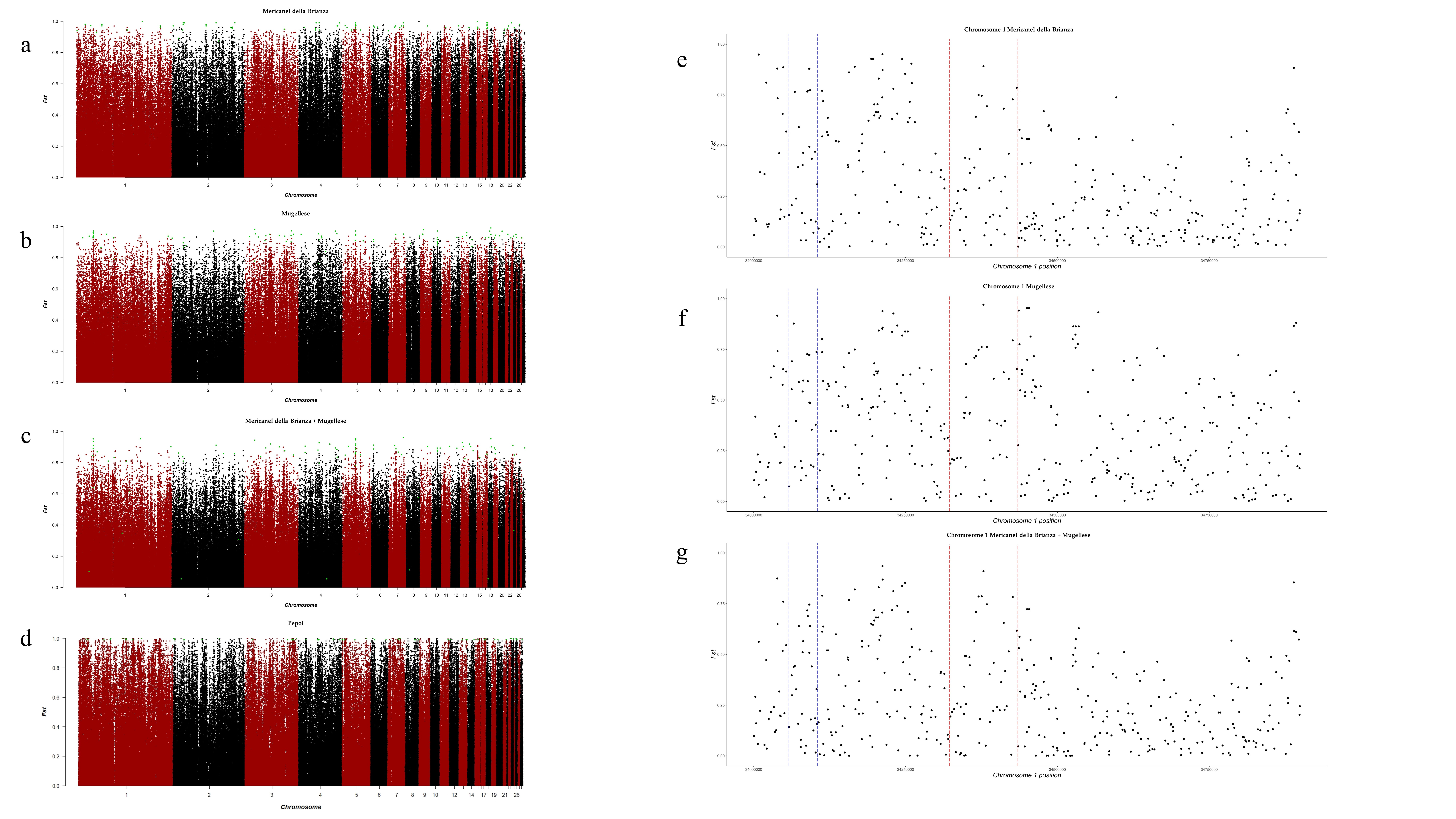

Supplement: Supplementary file 1 [file genes-14-00633-s001.zip › Supplementary Material Figure S3.jpg]

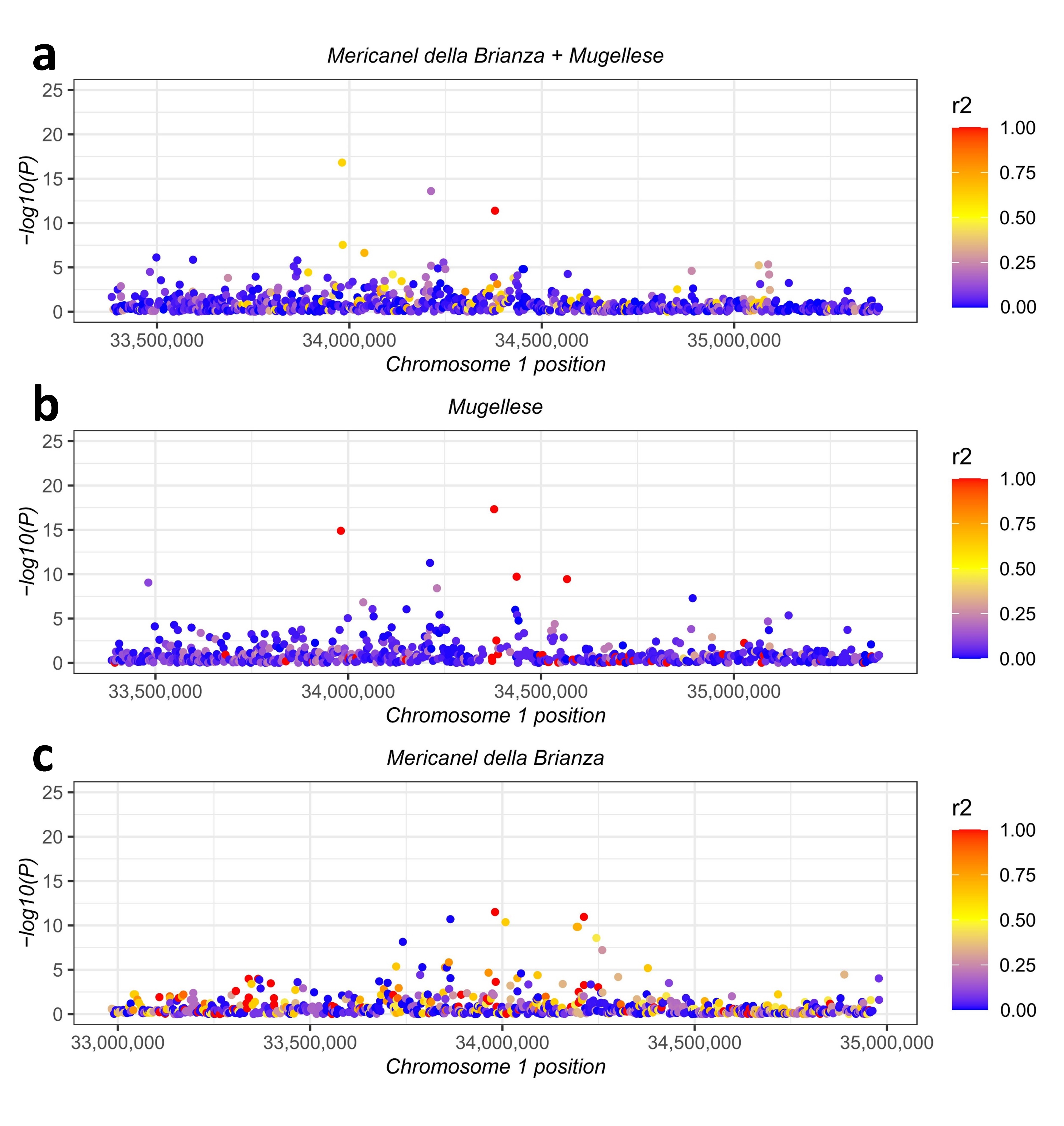

Supplement: Supplementary file 1 [file genes-14-00633-s001.zip › Supplementary Material Figure S4.jpg]

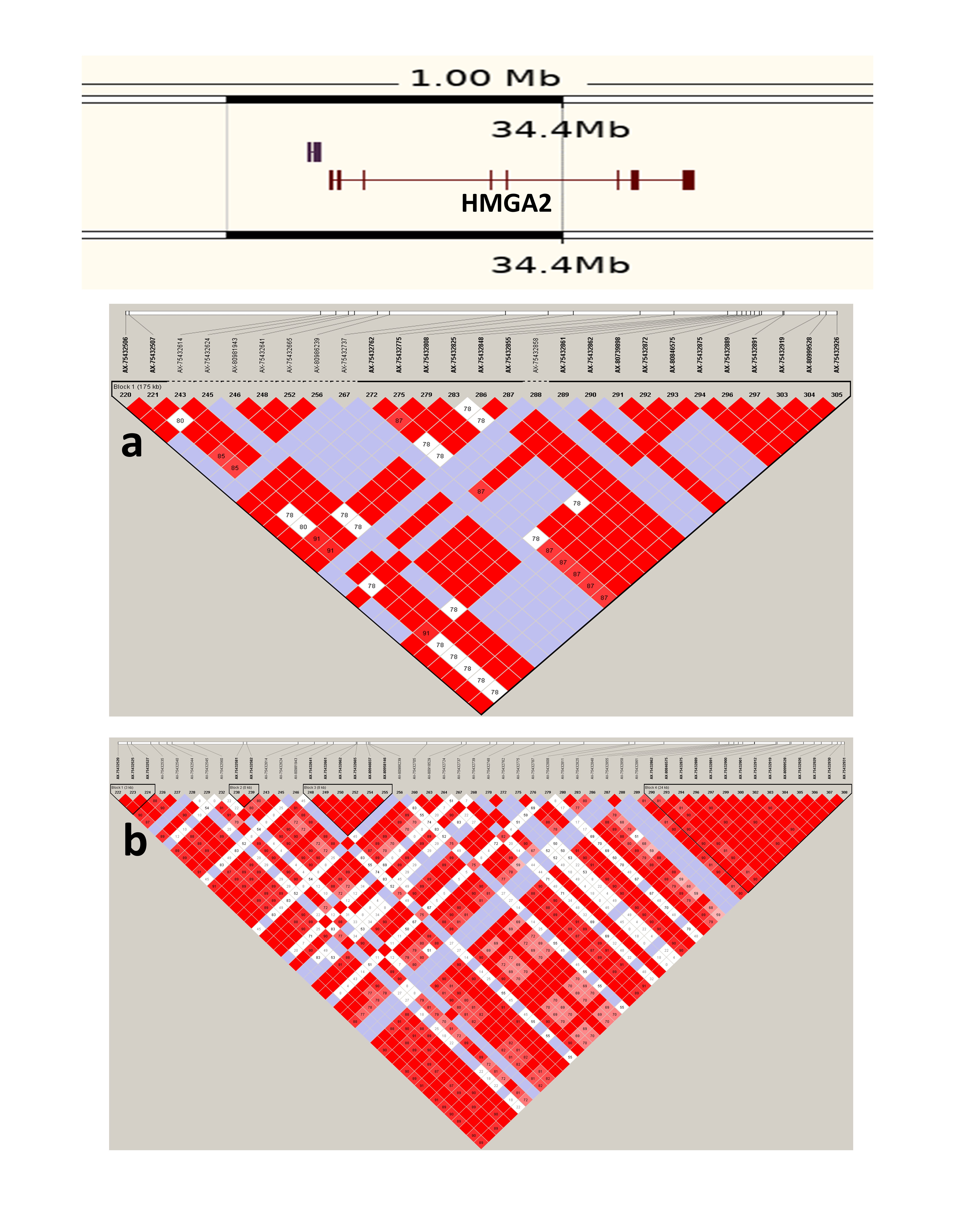

Supplement: Supplementary file 1 [file genes-14-00633-s001.zip › Supplementary Material Figure S5.jpg]
